# Episodic Sexual Transmission of HIV Revealed by Molecular Phylodynamics

**DOI:** 10.1371/journal.pmed.0050050

**Published:** 2008-03-18

**Authors:** Fraser Lewis, Gareth J Hughes, Andrew Rambaut, Anton Pozniak, Andrew J. Leigh Brown

**Affiliations:** 1 Institute of Evolutionary Biology, School of Biological Sciences, University of Edinburgh, Edinburgh, Scotland; 2 Chelsea and Westminster Hospital, London, United Kingdom; University of California San Francisco, United States of America

## Abstract

**Background:**

The structure of sexual contact networks plays a key role in the epidemiology of sexually transmitted infections, and their reconstruction from interview data has provided valuable insights into the spread of infection. For HIV, the long period of infectivity has made the interpretation of contact networks more difficult, and major discrepancies have been observed between the contact network and the transmission network revealed by viral phylogenetics. The high rate of HIV evolution in principle allows for detailed reconstruction of links between virus from different individuals, but often sampling has been too sparse to describe the structure of the transmission network. The aim of this study was to analyze a high-density sample of an HIV-infected population using recently developed techniques in phylogenetics to infer the short-term dynamics of the epidemic among men who have sex with men (MSM).

**Methods and Findings:**

Sequences of the protease and reverse transcriptase coding regions from 2,126 patients, predominantly MSM, from London were compared: 402 of these showed a close match to at least one other subtype B sequence. Nine large clusters were identified on the basis of genetic distance; all were confirmed by Bayesian Monte Carlo Markov chain (MCMC) phylogenetic analysis. Overall, 25% of individuals with a close match with one sequence are linked to 10 or more others. Dated phylogenies of the clusters using a relaxed clock indicated that 65% of the transmissions within clusters took place between 1995 and 2000, and 25% occurred within 6 mo after infection. The likelihood that not all members of the clusters have been identified renders the latter observation conservative.

**Conclusions:**

Reconstruction of the HIV transmission network using a dated phylogeny approach has revealed the HIV epidemic among MSM in London to have been episodic, with evidence of multiple clusters of transmissions dating to the late 1990s, a period when HIV prevalence is known to have doubled in this population. The quantitative description of the transmission dynamics among MSM will be important for parameterization of epidemiological models and in designing intervention strategies.

## Abstract 2

Using viral genotype data from HIV drug resistance testing at a London clinic, Andrew Leigh Brown and colleagues derive the structure of the transmission network through phylogenetic analysis.

## Editors' Summary

**Background.:**

Human immunodeficiency virus (HIV), the cause of acquired immunodeficiency syndrome (AIDS), is mainly spread through unprotected sex with an infected partner. Like other sexually transmitted diseases, HIV/AIDS spreads through networks of sexual contacts. The characteristics of these complex networks (which include people who have serial sexual relationships with single partners and people who have concurrent sexual relationships with several partners) affect how quickly diseases spread in the short term and how common the disease is in the long term. For many sexually transmitted diseases, sexual contact networks can be reconstructed from interview data. The information gained in this way can be used for partner notification so that transmitters of the disease and people who may have been unknowingly infected can be identified, treated, and advised about disease prevention. It can also be used to develop effective community-based prevention strategies.

**Why Was This Study Done?:**

Although sexual contact networks have provided valuable information about the spread of many sexually transmitted diseases, they cannot easily be used to understand HIV transmission patterns. This is because the period of infectivity with HIV is long and the risk of infection from a single sexual contact with an infected person is low. Another way to understand the spread of HIV is through phylogenetics, which examines the genetic relatedness of viruses obtained from different individuals. Frequent small changes in the genetic blueprint of HIV allow the virus to avoid the human immune response and to become resistant to antiretroviral drugs. In this study, the researchers use recently developed analytical methods, viral sequences from a large proportion of a specific HIV-infected population, and information on when each sample was taken, to learn about transmission of HIV/AIDS in London among men who have sex with men (MSM; a term that encompasses gay, bisexual, and transgendered men and heterosexual men who sometimes have sex with men). This new approach, which combines information on viral genetic variation and viral population dynamics, is called “molecular phylodynamics.”

**What Did the Researchers Do and Find?:**

The researchers compared the sequences of the genes encoding the HIV-1 protease and reverse transcriptase from more than 2,000 patients, mainly MSM, attending a large London HIV clinic between 1997 and 2003. 402 of these sequences closely matched at least one other subtype B sequence (the HIV/AIDS epidemic among MSM in the UK primarily involves HIV subtype B). Further analysis showed that the patients from whom this subset of sequences came formed six clusters of ten or more individuals, as well as many smaller clusters, based on the genetic relatedness of their HIV viruses. The researchers then used information on the date when each sample was collected and a “relaxed clock” approach (which accounts for the possibility that different sequences evolve at different rates) to determine dated phylogenies (patterns of genetic relatedness that indicate when gene sequences change) for the clusters. These phylogenies indicated that at least in one in four transmissions between the individuals in the large clusters occurred within 6 months of infection, and that most of the transmissions within each cluster occurred over periods of 3–4 years during the late 1990s.

**What Do These Findings Mean?:**

This phylodynamic reconstruction of the HIV transmission network among MSM in a London clinic indicates that the HIV epidemic in this population has been episodic with multiple clusters of transmission occurring during the late 1990s, a time when the number of HIV infections in this population doubled. It also suggests that transmission of the virus during the early stages of HIV infection is likely to be an important driver of the epidemic. Whether these results apply more generally to the MSM population at risk for transmitting or acquiring HIV depends on whether the patients in this study are representative of that group. Additional studies are needed to determine this, but if the patterns revealed here are generalizable, then this quantitative description of HIV transmission dynamics should help in the design of strategies to strengthen HIV prevention among MSM.

**Additional Information.:**

Please access these Web sites via the online version of this summary at http://dx.doi.org/10.1371/journal.pmed.0050050.

- Read a related *PLoS Medicine* Perspective article
- Information is available from the US National Institute of Allergy and Infectious Diseases on HIV infection and AIDS
- HIV InSite has comprehensive information on all aspects of HIV/AIDS, including a list of organizations that provide information for gay men and MSM
- The US Centers for Disease Control and Prevention provides information on HIV/AIDS and on HIV/AIDS among MSM (in English and Spanish)
- Information is available from Avert, an international AIDS charity, on HIV, AIDS, and men who have sex with men
- The Center for AIDS Prevention Studies (University of California, San Francisco) provides information on sexual networks and HIV prevention
- The US National Center for Biotechnology Information provides a science primer on molecular phylogenetics
- UK Collaborative Group on HIV Drug Resistance maintains a database of resistance tests
- HIV i-Base offers HIV treatment information for health-care professionals and HIV-positive people
- The NIH-funded HIV Sequence Database contains data on genetic sequences, resistance, immunology, and vaccine trials

## Introduction

Sexually transmitted infections spread through an often complex network of sexual contacts [1]. The characteristics of such a network play a vital role in determining both short-term dynamics and longer-term equilibrium prevalence of disease [2,3]. Early epidemiological modelling of the HIV epidemic among men who have sex with men (MSM) identified primary infection, when infectivity might be highest [4], as a potential driver of the epidemic [5], but empirical evidence to support this has been lacking. The reconstruction of contact networks from interview data has provided valuable insights into epidemics of sexually transmitted infections such as *Chlamydia* [6] and gonorrhoea [7], and has been applied in studies of some HIV-infected populations [8,9]. However, the interpretation of the contact network in the context of HIV infection is more problematic because of the long period of infectivity and the low average risk of infection per contact [10]. Although in some cases it has been shown that the reconstructed HIV transmission network maps closely onto the contact network [11,12] in other cases the transmission network was not reflected by the contact network [13,14].

Another line of investigation has taken the approach of phylogenetic analysis of population-based samples of viral sequences. These have yielded different outcomes according to risk group. Infections among injection drug user populations often reveal clustering to a greater or lesser extent [15–17], associated with a pattern of explosive epidemics among this risk group [18,19]. In contrast, evidence for infection clusters from analysis of sequences from population surveys of individuals infected by sexual contact is quite limited, and the typical structure of a phylogeny of viral sequences from such a population is star-like [15]. The United Kingdom MSM HIV epidemic is a subtype B epidemic and is rooted in that of the United States [20], probably with multiple introductions into the UK population [21]. Even though some studies have been predicated on the higher infectivity of individuals in acute infection [4], these have usually nevertheless identified a limited number of such networks of rather low degree (connectivity) [22]. In one such recent study, a larger number of clusters were described [23], but this was from a population of acutely infected individuals that included a high proportion of injection drug users (34% of total); only 54% were MSM [24]. The extent to which sexual transmission of HIV among MSM is clustered therefore remains open.

One possible reason why uncertainty over the degree of clustering has persisted has been the nature of sampling. Diagnosis of acute (or recent) infection is usually made in only a small proportion of individuals. Pilcher et al. describe 107 (18%) and 23 (4%) individuals out of 583 as “recent” and “acute” infections, respectively [25]; Pao et al. similarly report that 103 recent infections (8%) were identified out of 1,235 patients seen [22]. Population-based analyses in chronic infection have in the past been restricted in scale for computational reasons to low-density samples [15]. Such sampling will inevitably bias the results towards under-reporting of clusters, especially those of higher degree.

There have been a number of recent developments that have permitted a new approach to this issue. The recommendation that patients with HIV should receive a genotype test for resistance before commencing antiretroviral therapy (ARV) [26] has led to a substantial increase in the availability of viral sequence data from HIV-infected individuals. Continual improvements in computational resources have allowed previously unapproachable datasets to be analysed, and new analytical methods have been developed which incorporate sample date information and allow the evolutionary dynamics of a population to be inferred from sequence data. Such approaches, collectively termed “phylodynamics,” have been applied to a number of infectious agents because of the availability of datasets of sequences from dated samples and the rapid rate of evolution of RNA viruses [27].

In this study, we have used HIV sequences generated from routine clinical treatment to provide a dense sample of the population attending a large London clinic. We adopted a “relaxed clock” [28] methodology to analyse these sequences in order to infer the molecular phylodynamics of the HIV epidemic among MSM in London.

## Methods

### Dataset

Our base dataset comprised 2,126 anonymised HIV-1 nucleotide sequences (concatenated full-length protease [PR] and partial reverse transcriptase [RT] coding sequences, 1,497 nucleotides in length) from patients attending HIV clinics at the Chelsea and Westminster Hospital, London, during the period 1997–2003. The Chelsea and Westminster clinic is the largest clinic serving patients with HIV in London, with more than 6,500 patients, contributing 29% of the London patients to the United Kingdom Collaborative HIV Cohort (UK CHIC) study in 2006. Its primary catchment area comprises the inner-city London boroughs of Westminster, Kensington and Chelsea, and Wandsworth, with an HIV-infected patient population that is characteristic of London, including a high proportion (>75%) of MSM. Sequences were provided by VircoBVBA (Michelen, Belgium), having been generated for VircoGEN resistance reports for patients about to start therapy or experiencing failure of therapy. Overall, 384 (18%) patients were receiving ARV at the time the analyzed sample was taken. More details of patients receiving ARV are given in Table S1. Ethical approval for this work was given by the London Multicentre Research Ethics Committee (MREC/01/2/10; 5 April 2001).

For patients with multiple sequences, only the oldest sequence was included. Sex and self-reported exposure group were available for each sequence, but other identifiers and clinical data were delinked to maintain confidentiality. HIV-1 subtype was determined using the REGA HIV Subtyping Tool version 2.0 [29].

### Identification of Transmission Clusters

In order to remove the influence of convergent evolution at antiretroviral drug resistance mutations on the phylogenetic analysis, two versions of the dataset were analyzed: (i) third-base positions only (for analyses of exact and exact plus ambiguous differences; 499 sites) and (ii) a codon-stripped dataset from which 37 codons associated with major resistance in PR (30, 32, 33, 46, 47, 48, 50, 54, 76, 82, 84, 88, and 90) and RT (41, 62, 65, 67, 69, 70, 74, 75, 77, 100, 103, 106, 108, 115, 116, 151, 181, 184, 188, 190, 210, 215, 219, 225, and 236) were stripped from the alignment (leaving 1,386 nt). Analyses based on genetic distance made use of the first dataset using uncorrected (Hamming) distances; those based on Bayesian Monte Carlo Markov chain (MCMC) phylogenetic approaches used the second.

Phylogenies were constructed with MrBayes [30] using a general time-reversible (GTR) model of nucleotide substitution with a proportion of invariant sites (ι) and gamma distribution of rates (Γ). The MCMC search was run for 5 × 10^6^ generations, with trees sampled every 100th generation (with a burn-in of 50%) and a posterior consensus tree generated (from 25,000 trees). From this consensus tree, the posterior probability of nodes was used as phylogenetic support for each transmission cluster group. Cluster group size was determined using nodes with a posterior probability of 1.

### Ancestral State Reconstruction

The ancestral state of amino acids for each cluster group was determined using MrBayes. Separate runs (10^6^ generations, sampling every 100 generations, burn-in 25%) were performed for each group using an HIV-1 subtype C sequence as outgroup under the GTR + I + Γ model of nucleotide substitution. For each run, trees were constrained to include a topology prior for a monophyletic group comprising the cluster group of interest. At the root node of this constraint, the ancestral states of each of the 37 amino acid positions (both genes) associated with drug resistance were compared to known mutations attributed to drug resistance (http://www.iasusa.org/).

### Time-Scaled Phylogenies

Dated phylogenies were obtained using a Bayesian MCMC method (BEAST version 1.4.2; available from http://beast.bio.ed.ac.uk/ [28]). The date used for each sequence in any analysis was the number of days since the isolation date of the oldest sequence, with sequences dated using the number of days from the earliest sequence isolation date. Separate analyses were performed using the GTR + I + Γ and an adaptation of the SRD06 nucleotide substitution models [31]. The adapted SRD06 model used two independent relaxed molecular clocks for first/second and third codon positions. For each analysis, a MrBayes phylogenetic tree was generated and used as a starting tree for BEAST. The most appropriate demographic model (either a constant or exponential model of population size) and distribution of rates amongst branches (lognormal or exponential) were determined using the GTR + I + Γ model. Log-normal priors were placed on root height (under the assumption that individual clusters were likely to have formed within 10 y [median of the distribution] of the latest sequences but could have formed within 40 y [95% upper limit]), population size (assuming a median value of effective population size that approximated that of the size of the individual clusters), and a Jeffrey's prior placed on mean substitution rate. For each cluster group, the selected model was used for two separate MCMC runs of chain length 5 × 10^6^ (sampling parameters and trees every 1,000 generations). All parameters were estimated from an effective sampling size >200. Trees generated from both BEAST runs were combined and trees summarized after a 10% burn-in (leaving 9,000 trees) using TreeAnnotator (available from http://beast.bio.ed.ac.uk). As external branches represent the termini of transmission chains, analysis was restricted to internal branch lengths extracted from time-scaled BEAST consensus trees.

## Results

### Selection of the Transmission Network

We analyzed consensus sequences of the HIV-1 PR and RT coding regions obtained from the plasma viral population of patients (*N* = 2,126) attending HIV clinics at the Chelsea and Westminster Hospital, London, between 1997 and 2003. As expected of sequences obtained during chronic HIV infection, these were highly variable, with an average (uncorrected) difference between sequences of 12% at synonymous sites among the 1,695 subtype B sequences (Table S2). To maximize the information used in initial comparisons between virus sequences from different individuals, we made use of ambiguous as well as exactly identified nucleotides. Ambiguous nucleotides (denoted by IUPAC symbols; e.g., R = G or A and M = A or C) occur frequently in consensus HIV sequences because of the presence of co-circulating variants within patient plasma samples. We scored an exact difference at a site where there was no overlap (e.g., A versus T or M versus T), and an ambiguous difference (e.g., M versus A or M versus R) if overlap was identified. Exact differences between patient consensus sequences represent the fixation of different alleles in the respective viral populations, while ambiguous differences reflect polymorphic sites where they still share alleles. As evolutionary divergence between populations increases, the number of sites where alleles are shared decreases. We therefore expect a negative slope when these values are plotted against each other.

**Figure 1:**
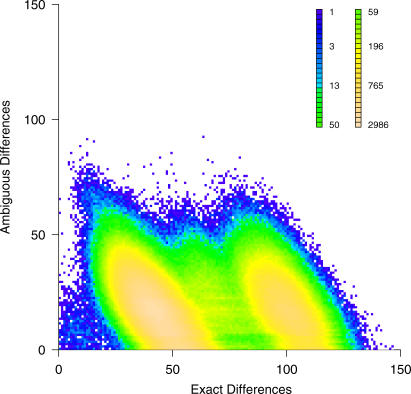
Distribution of Genetic Distance Among All Pairwise Comparisons of 2,126 Patient-Derived HIV Sequences Key indicates the number of comparisons for each datapoint by colour. Sequences were compared at all 499 third-base sites and recorded for an exact difference or an ambiguous difference (see text for details). Two major peaks reflect within subtype (30–60 differences) and between subtype (100–110 differences) comparisons, respectively. The third smaller region of density close to the origin identifies patients with at least one other closely related sequence in the dataset.

Initial comparisons of all sequences in the dataset required a simple, computationally tractable approach because of its large size (2.26 × 10^6^ pairwise comparisons), while recognizing the potential for bias through convergent evolution in patients prescribed the same drugs. This was avoided by restricting analysis to the third-base position in the 499 codons sequenced. We present these data as a colour density graph of the number of exact and ambiguous differences between all patient consensus sequences (Figure 1). Two major density peaks are observed, both showing the expected negative slope. As this is a diverse patient population with multiple HIV-1 subtypes, the density peak with the higher number of differences can be interpreted as corresponding to inter-subtype comparisons, and that with the smaller number to intra-subtype comparisons (Figure 1).

In addition to the two major peaks, a small, yet distinct, third density peak can be seen close to the origin, which represents pairwise comparisons between particularly closely related sequences. The apparent trough in density between this and the adjacent group corresponded to approximately 25 nucleotide differences, which was used to define a subset of sequences that had at least one other close relative. This subset includes 483 patients, of which 402 were infected with HIV-1 subtype B. These 402 were overwhelmingly MSM, with just ten self-reporting injection drug use (four female, six male), and 13 self-reporting heterosexual contact (nine female, four male), as a risk factor, and were the basis of the detailed studies described below.

### Epidemiological and Phylogenetic Structure of the Transmission Network

**Figure 2:**
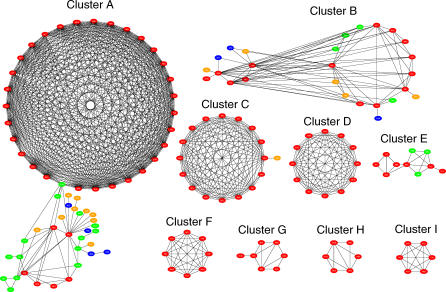
HIV Transmission Clusters Defined by Genetic Distance Patients included in major clusters are represented by a red node, and connecting lines between red nodes represent a genetic distance of less than 4.8% (24 differences). Sensitivity of the clusters to the distance criterion shown by additional nodes in green (5.0%), blue (5.2%), and orange (5.4%).

In order to identify patterns of transmission among the 402 subtype B sequences, two complementary approaches were adopted. The first, based on the matrix of pairwise exact differences at 499 third-base positions, recognized all linkages between individuals involving 24 differences (4.8%) or fewer. Many of the groups revealed link only two or three individuals, but nine groups with six or more members were identified using this criterion (Figure 2). These largest groups linked 30, 16, 14, 12, eight, seven (×2), and six (×2) individuals each. As a sensitivity analysis, the criterion was relaxed successively to 25, 26, and 27 differences (5.0%, 5.2%, and 5.4%, respectively), and the change in group was size noted (Figure 2). Only two groups changed size substantially, indicating discontinuities in the difference matrix. Cluster A (30 members at 24 differences) grew to 63 members at 27 differences, and Cluster B (14 members) to 26. Clusters D (12 members), F (eight members), G (seven members), H (six members), and I (six members) did not change, while cluster C (16 members) added one and E grew from six to nine members (Figure 2). Overall, there was a surprising degree of consistency at different thresholds, suggesting that the clusters identified represented distinct, bounded, epidemiological entities.

**Figure 3:**
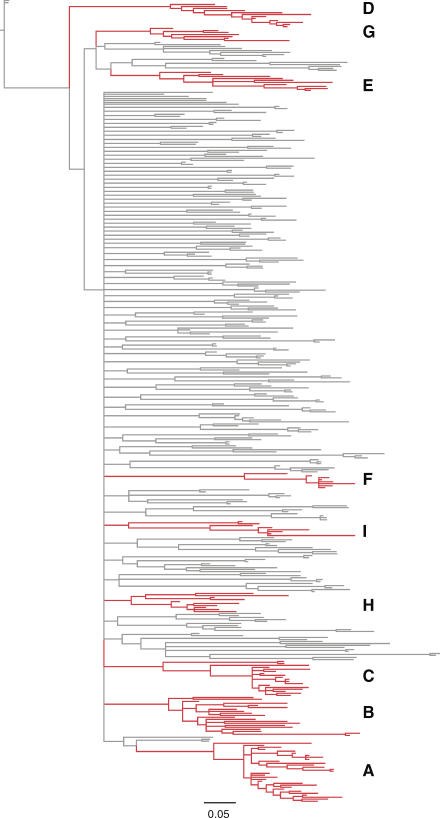
Bayesian MCMC Phylogenetic Tree of All Sequences Closely Linked to At Least One Other (*N* = 402) Clusters with ≥10 members (and a posterior probability of 1) are shown in red. Letters indicate the position of identified clusters. Scale bar indicates number of substitutions.

**Figure 4:**
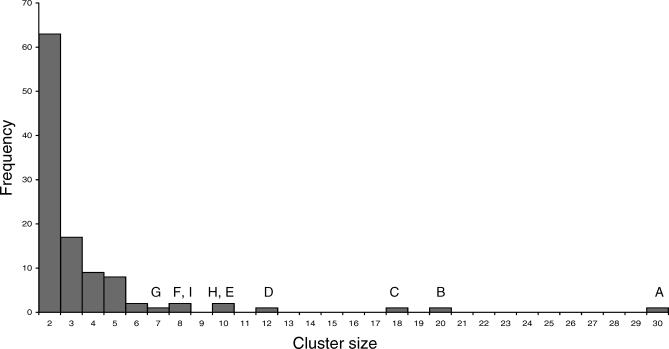
Distribution of Cluster Size from MrBayes Phylogenetic Tree of Closely Related Sequences (*N* = 402) A cluster is defined by nodes with a posterior probability of 1. Letters indicate the six largest clusters phylogenetically defined.

In the second approach, we performed a detailed phylogenetic analysis on the same sequence dataset. To maximize resolution, the full sequence dataset was used (all codon positions), but with 37 codons associated with major resistance in PR and RT removed, leaving 1,386 aligned nucleotide positions. A Bayesian MCMC phylogeny was reconstructed from these site-stripped sequences, using a subtype C sequence as an outgroup (Figure 3). In this approach, we define clusters as clades identified with a posterior probability of 1. All the clusters identified using the genetic distance approach meet this criterion on the Bayesian MCMC tree (Figure 3). Differences between them relate to the size of the clusters identified, usually due to the inclusion of a small number of additional sequences when defined phylogenetically. The overall distribution of cluster size under this definition is shown in Figure 4. From this it can be seen that of all patients with a close link to one other sequence in the database, the great majority (85%) have links to more than one, and 25% are closely associated with 10 or more others.

Six of the larger clusters were made up entirely of MSM (clusters B, D, E, F, H, and I). Clusters A and C are also primarily composed of MSM, but include one female patient in each. Cluster G (seven individuals) included one female and one injection drug user. Thus, the clustering identified in this study reflects the epidemiology of HIV transmission by sexual contact among MSM.

### Detailed Phylogenies of Clusters

**Figure 5:**
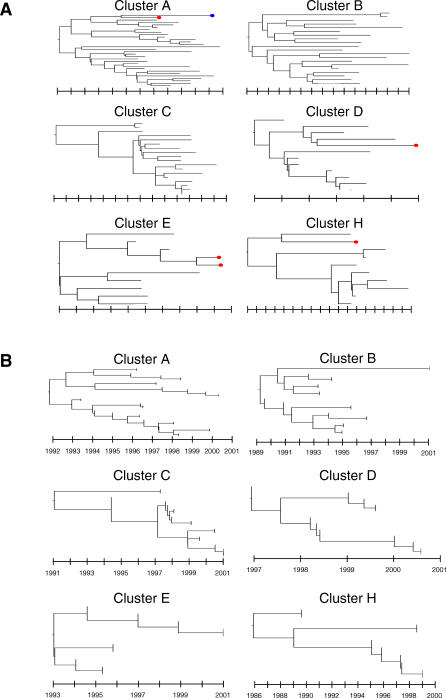
Relaxed-Clock Time-Scaled Phylogenies for the Six Largest Clusters Time-scaled phylogenies were generated using the partitioned SRD06 model. (A) Full trees with scale bar graduations in years. (B) Terminal branches removed with scale in calendar years to show timing of transmission events.

Clusters A–E and H, comprising 88 patients in all, were selected for further analysis on an individual basis using phylogenetic methods incorporating a relaxed molecular clock (the other clusters were not large enough for this analysis). For each cluster, an exponential model of population growth was significantly favoured over a constant population size (data not shown). Evolutionary rate estimates were obtained using the unpartitioned (GTR + I + Γ) nucleotide substitution model and the SRD06 model, which has two rate partitions, for third-base position and first- plus second-base positions respectively. Substitution rates at third-base positions vary nearly 3-fold among clusters (Figure S1), but the differences are not significant. The highest mean rate estimate at third-base positions was 4 × 10^−4^ for cluster D, while the mean rate at first- and second-base positions varied between 5 × 10^−5^ for cluster C and 1 × 10^−4^ (cluster D). The coefficient of variation in substitution rate was 0.6 or less for all clusters except cluster A, the largest cluster, which was 1.12 (Table 1). On the basis of 95% highest probability distributions, a log-normal rate distribution was adopted for all clusters to generate time-scaled trees (Figure 5A). Each of the clusters reveals some degree of internal structure, particularly the two largest clusters, A and B. In these it can be seen that there are two subclades in cluster A comprising sequences that are much more closely related to each other than they are to those of the other.

**Table 1:**
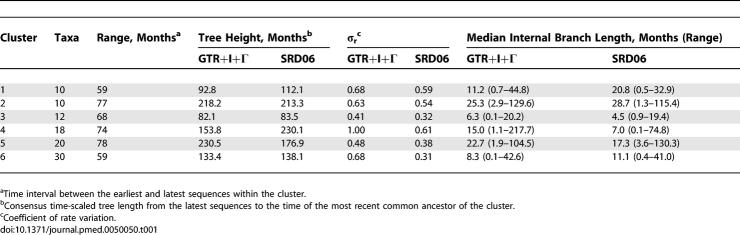
Relaxed-Clock Analysis of Major Clusters Using BEAST

### Distribution of ARV Resistance–Associated Mutations

It is important to know whether the structure of the transmission network has influenced the transmission of drug-resistant virus. We have identified all patient samples with resistance-associated mutations in the six clusters by coloured circles at the tips (Figure 5A). There are a total of six patients in four clusters, five with mutations in RT and 1 with mutations in PR (Table S3). In one case in cluster A and a second in cluster E, two patients who were nearest neighbours in the phylogeny both had resistance-associated mutations. However, the mutations are either entirely (cluster A) or substantially (cluster E) different in the patient pairs (Figure S3), so there is no clear evidence of transmission of drug resistance in any of these clusters.

### Dating Transmission Events

Each viral sequence in the time-scaled phylogenies represents a different patient. Therefore, for any two sequences, the branches connecting them through their most recent common ancestor (MRCA) include at least one transmission event. Consequently, the distance between the MRCA and the previous node estimates the upper bound of time between transmission events. Figure 5B shows the structure of the time-dated phylogenies with tips removed, revealing the variation in internode distances. This representation is scaled by calendar year, from which we can infer the periods over which these clustered transmissions occurred. For clusters A and E this spans much of the 1990s, from 1994 to 2000, while the transmissions that link cluster B occurred earlier in the decade, up to 1995. Most transmissions in the remaining clusters (C, D, and H) occurred in the latter part of the decade. The procedure used to estimate the MRCA dates allows us to provide an indication of the confidence in these dates through the marginal distributions of the times to the MRCA (TMRCA) for the subclades (Figure S4). As expected, these are always narrowest for the most recent nodes and become much shallower as the time between the node and the dated samples increases.

**Figure 6:**
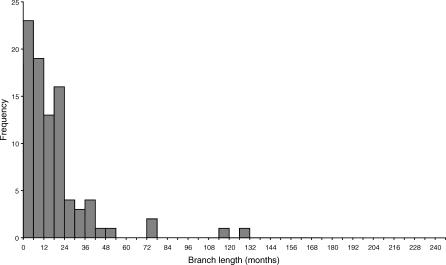
Histogram of Internal Branch Lengths (*N* = 88) from All Six *N* ≥ 10 Clusters Estimated Using the Partitioned SRD06 Model Median branch length was 13.14 mo, and the 25th percentile was 5.8 mo. Similar results were obtained using the GTR + I + Γ model (unpublished data).

It is very likely that not all members of any transmission cluster have been sampled, so the average time between transmissions will almost certainly be overestimated. Analysis of the overall distribution of internode intervals, estimated from the trees (Figure 6), reveals a median of 14 mo, but is highly skewed so that 25% of internode intervals were of 6 mo or less. As the patients in these clusters represent 25% of those with at least one linked sequence, this suggests that at least 5% of transmissions on average occur within 6 mo of infection in this almost exclusively MSM population.

## Discussion

We have made use of sequence data obtained for resistance genotyping for the largest clinical centre treating patients with HIV in London to reconstruct the transmission network in this population. In contrast to previous studies based on sparsely sampled populations, by examining all pairwise comparisons among sequences from more than 2,000 patients, we were able to identify a subset of 402 subtype B pairs with a genetic distance of 5% or less. Detailed phylogenetic analysis identified a number of large clusters among these patients, which together comprised 25% of this group. “Dated phylogeny” analysis of these clusters [28] revealed an episodic pattern, with many of the transmissions within them occurring within a short space of time.

New HIV diagnoses among MSM have risen steadily in the United Kingdom for almost 10 years, and are now approaching twice the number recorded annually in the mid to late 1990s [32]. Efforts to characterize the changes in this population that have been responsible, including the National Survey of Sexual Attitudes and Lifestyles (NATSAL; [33]), have provided substantial amounts of information on current risk behaviour of this population. Unprotected anal intercourse with one or more partners in the past year was reported by between 32% and 45% of MSM [34] recruited in different surveys; approximately 18% of respondents in another study reported unprotected anal intercourse with individuals of unknown HIV status [35], and 3.2% of respondents reported unprotected anal intercourse with five or more partners in the previous year [34]. There was also a notable and significant increase in prevalence of risk activities between the 1990 and 2000 NATSAL surveys [36].

We have used a database of HIV sequences collected in the course of routine clinical treatment from 2,126 patients to characterize the relationships between viruses infecting different individuals attending a large clinic in London. The depth of sampling meant this study was much more informative about the transmission patterns than previous studies [15]. We identified 402 individuals whose virus had a close relationship with at least one other, and using two different approaches showed that almost 90 of these individuals could be linked in clusters of 10 or more individuals. Using information on the date each sample was taken, we have reconstructed dated phylogenies that revealed that at least 25% of transmissions among these individuals occurred within a few months of their infection. The tightness of clustering is striking, with most of the linked transmissions occurring within periods of at most 3–4 y. The closeness of these events is inevitably underestimated as a result of incomplete data (i.e., intervening individuals not sampled), so the actual average time between transmissions in these clusters is likely to be smaller. Extrapolation of the conclusions from this clinic population more widely depends on the degree to which patients attending the Chelsea and Westminster clinic reflect the UK MSM population with HIV as a whole. This clinic is the largest HIV clinic in the UK and has contributed 6,551 (24%) out of a (2006) total of 26,811 patients to the UK CHIC study [37], comprising 29% of all the patients from London. While the location of its primary catchment area within central London suggested it would be likely to be representative, we have recently been able to extend these studies to the entire UK CHIC patient population. Preliminary analysis of HIV genotypes from 8,088 patients that have been investigated for clustering using the genetic distance approach revealed 2,150 individuals with a link to at least one other patient. Among these, several large clusters have been observed, with Chelsea and Westminster patients distributed among patients from other clinics (data not shown). We are therefore confident the pattern described in the current study reflects that of the wider UK population of MSM with HIV.

Other possible limitations of the study should be recognised. Although the use of a phylogenetic definition of clusters avoids the necessity to select an arbitrary distance value, there are clear restrictions on what can be concluded from the phylogeny. The similarity between many of the sequences *within* these clusters is frequently so high that there is little power to estimate the internal order of transmissions with any confidence. Neither is it possible, from the phylogeny alone, to determine direction of transmission, or how many individuals in the cluster were transmitters. Thus, cluster E (Figure 5) could have been generated by transmissions from a minimum of three individuals transmitting to six, one, and two others, respectively; or alternatively, from a maximum of seven, where one transmits to three others and all others transmit once. The former situation would be expected under a more skewed distribution of partner numbers, which has been suggested by several studies [8,33,34,38]. These results therefore complement rather than replace studies that would define parameters such as partner number.

One of the possible consequences of rapid transmission within clusters is a local increase in the transmission of drug-resistant strains [39,40]. Whether this had occurred was examined by maximum likelihood reconstruction of ancestral states at all sites associated with drug resistance in the six large clusters. In no case was a drug-resistant virus identified at the root of these clusters (unpublished data), which may have reflected the time at which these particular events were occurring (Figure 5B). The distribution of mutations at the tips of the trees also do not suggest extensive transmission of resistance-associated mutations. There were only two cases where nearest-neighbour patients both had mutations, and the mutations differed between the pair in each case, suggesting they were all examples of secondary rather than primary resistance.

As the time-dependent phylogenies are calibrated in calendar years, we are able to estimate when most of the transmissions in each cluster occurred. For cluster A, the largest cluster comprising 30 individuals, most of the transmissions between them occurred within about 6 y preceding 1999 (Figure 5B); for cluster C, the transmissions were tightly restricted to 1995–1997, with many estimated to have occurred in 1996; for cluster B, however, the transmissions mostly occurred between 1991 and 1995. Many of the transmission intervals (with the exception of cluster B) lie within or closely precede the period during which HIV diagnoses have been increasing. Given an expected delay from infection to diagnosis of 3–5 y, we could infer that these clustered transmissions contributed to the increase in prevalence that occurred in London and the United Kingdom in that time [32]. From these results we can also say that many of these transmission clusters were initiated relatively early in the highly active antiretroviral therapy (HAART) era and before transmitted ARV resistance became a significant problem in the UK [40].

As epidemiological models become increasingly complex, incorporating variable mixing patterns [41], quantitative data on the transmission network structure and the dynamics of transmission will be vital to ensure appropriate parameterization. The level of epidemiologically relevant information yielded by the time-dated phylogeny with respect to the structure and dynamics of the HIV transmission network in this population represents a substantial increase in our depth of knowledge on which interventions can be based.

## Supporting Information

Figure S1 Nucleotide Substitution Rates Estimated from BEAST Rates estimated using the GTR + I + Γ (solid line) and SRD06 (empty square = third-codon position; filled circle = first-/second-codon positions) models, respectively for the six largest clusters. Error bars show 95% highest posterior density of SRD06 rates.
(34 KB PPT)

Figure S2 Distribution of Internal Branch Lengths Estimated from BEAST Rates using the GTR + I + Γ (filled circles) and SRD06 (empty circles) substitution models are shown separately (solid line = median) for the six largest clusters.
(55 KB PPT)

Figure S3 Identification of Antiretroviral Resistance–Associated Mutations on the Cluster Phylogenies (115 KB PPT)

Figure S4 Dated Phylogenies with Marginal Distributions for TMRCAs BEAST analysis is based on a summary of a large number of phylogenetic trees where individual trees may have a slightly different topology (depending on the degree of support for the existence of each node). To indicate support for an estimated node date, it is possible to estimate the marginal distribution of TMRCA for all taxa that are found within below each node of the maximum clade credibility tree. For each node within each cluster tree, the distribution of the TMRCA was calculated from the same tree sample used to produce the trees. The distributions of each TMRCA have been aligned (according to time) with the cluster trees from Figure 5.
(1.5 MB PPT)

Table S1 Proportion of Patients Receiving ARV at the Time of First Sampling Numbers of analyzed samples where patient had been receiving ARV before sample was taken, by year.
(30 KB DOC)

Table S2 Genetic Variation in the PR and RT Domains Among 1,695 Subtype B Sequences from the Chelsea and Westminster Clinical Cohort Average difference between 1,695 subtype B sequences and proportion of polymorphic sites. p-distance: uncorrected genetic distance; dS p-distance: uncorrected distance at synonymous nucleotide sites; dN p-distance: uncorrected distance at non-synonymous nucleotide sites.
(27 KB DOC)

Table S3 Details of Resistance Mutations Detected Within Clusters Numbers of resistance-associated mutations observed in each cluster according to coding region and in total.
(38 KB DOC)

### Accession Numbers

The sequences analyzed have been deposited in GenBank (http://www.ncbi.nlm.nih.gov/Genbank) under accession numbers EU236439–EU236538. The GenBank accession number for the HIV-1 subtype C sequence is AF110959.

## Abbreviations

ARV: antiretroviral therapy
GTR: general time-reversible
MCMC: Monte Carlo Markov chain
MRCA: most recent common ancestor
MSM: men who have sex with men
PR: protease
RT: reverse transcriptase
TMRCA: time to MRCA
UK CHIC: United Kingdom Collaborative HIV Cohort
