# Supplementary material for: Episodic Sexual Transmission of HIV Revealed by Molecular Phylodynamics
Source: PLoS Med. 2008 Mar 18;5(3):e50. doi: 10.1371/journal.pmed.0050050 (PMC2267814; doi:10.1371/journal.pmed.0050050)
Supplement: Supplementary file 1 [file pmed.0050050.st001.doc]

**Supporting Information**

|  | 1997 | 1998 | 1999 | 2000 | 2001 | 2002 | 2003 | Total | % |
| --- | --- | --- | --- | --- | --- | --- | --- | --- | --- |
| ARV | 42 | 61 | 55 | 42 | 58 | 74 | 52 | 384 | 18 |
| No ARV | 371 | 280 | 210 | 253 | 312 | 210 | 106 | 1742 | 82 |
| Total | 413 | 341 | 265 | 295 | 370 | 284 | 158 | 2126 | 100 |
| % | 10 | 18 | 21 | 14 | 16 | 26 | 33 |  |  |

Table S1.
