# Supplementary material for: Episodic Sexual Transmission of HIV Revealed by Molecular Phylodynamics
Source: PLoS Med. 2008 Mar 18;5(3):e50. doi: 10.1371/journal.pmed.0050050 (PMC2267814; doi:10.1371/journal.pmed.0050050)
Supplement: Supplementary file 2 [file pmed.0050050.st002.doc]

| *Gene* | *Length (nts)* | *p-distance* | *dS p-distance* | *dN p-distance* | *Polymorphic sites* |
| --- | --- | --- | --- | --- | --- |
| PR | 297 | 0.049 | 0.103 | 0.032 | 224 (75%) |
| RT | 1200 | 0.046 | 0.133 | 0.023 | 857 (71%) |
| Both | 1497 | 0.047 | 0.127 | 0.024 | 1081 (72%) |

Table S2
