# Supplementary material for: Episodic Sexual Transmission of HIV Revealed by Molecular Phylodynamics
Source: PLoS Med. 2008 Mar 18;5(3):e50. doi: 10.1371/journal.pmed.0050050 (PMC2267814; doi:10.1371/journal.pmed.0050050)
Supplement: Supplementary file 3 [file pmed.0050050.st003.doc]

| *Cluster* | *N* | *Sequences with resistance mutations* | | | *Mutation counts for sequences, PR/RT* | *Total resistance mutations* | |
| --- | --- | --- | --- | --- | --- | --- | --- |
|  |  | *PR* | *RT* | *Any* |  | *PR* | *RT* |
| 1 | 10 | 0 | 2 | 2 | 0/2, 0/3 | 0 | 5 |
| 2 | 10 | 0 | 1 | 1 | 0/3 | 0 | 3 |
| 3 | 12 | 0 | 1 | 1 | 0/1 | 0 | 1 |
| 4 | 18 | 0 | 0 | 0 | - | 0 | 0 |
| 5 | 20 | 0 | 0 | 0 | - | 0 | 0 |
| 6 | 30 | 1 | 1 | 2 | 1/0, 0/1 | 1 | 1 |
| **Total** | **100** | **1** | **5** | **6** | **-** | **1** | **10** |

Table S3
